# Effects of creativity on social and behavioral adjustment in 7‐ to 11‐year‐old children

**DOI:** 10.1111/nyas.13944

**Published:** 2018-08-05

**Authors:** Daisy Fancourt, Andrew Steptoe

**Affiliations:** ^1^ Department of Behavioural Science and Health University College London London UK

**Keywords:** creativity, social adjustment, behavioral adjustment, children, depression, maladjustment

## Abstract

This study sought to explore whether creativity in undertaking activities such as free writing, telling a story, crafts, painting, drawing, or drama at age 7 is associated with a lower risk of social and behavioral maladjustment in children at the onset of adolescence. Data from 7558 7‐year‐olds who were socially and behaviorally “stable” at baseline were analyzed from the nationally representative National Child Development Study. Multinomial regression analyses showed associations between teacher‐rated creativity at age 7 and a lower relative risk of social and behavioral instability and maladjustment at age 11. Specifically, the associations were found between moderate and marked creativity and a lower risk of symptoms of internalizing behaviors (including depression and withdrawal), externalizing behaviors (such as restlessness) as well as a lower risk of various nervous symptoms of social and behavioral instability and maladjustment. Associations were independent of social, demographic, educational, parental, academic, and personality covariates, and robust to a range of sensitivity analyses. These results suggest that facilitating engagement with creative activities could be explored further as a way of reducing levels of instability and maladjustment at the onset of adolescence.

## Introduction

There are many well‐known case studies of individuals who display high levels of “creativity” alongside emotional instability, personality conflicts, and mania, which has led to a common public perception that creativity and mental illness and maladjustment are linked.^1^ However, there are several challenges to this. First, creativity is a broad construct, which can include seeing creativity as a personality trait, a cognitive process or a product.^2^ The creative genius of a selected few individuals who make remarkable discoveries and inventions (also known as “Big C” creativity) should be distinguished from everyday creative thoughts and acts (also known as “little c” creativity) that are thought to be visible from approximately the age of 2 and develop across the lifespan through engagement with imaginative activities and play.^3^ Second, for neither of these two types of creativity does evidence suggest an association with mental illness. Indeed, for “Big C” creativity, meta‐analyses of studies have found inconsistent results.^4^ And for “little c” creativity, research to date suggests that creativity may in fact be protective against mental illness. For example, a study of 24 creative and 24 noncreative seventh‐grade children found that those who were creative were less anxious.^5^ Creativity in taking part in artistic activities (such as painting, dancing, and storytelling) at age 7 in conjunction with intelligence and parental involvement with children has been identified as a risk‐reducing factor for the development of malaise and schizophrenia at the onset of puberty and in later life.^6^, ^7^, ^8^ And test scores for creative thinking (fluency, flexibility, and originality) are negatively correlated with maladjustment and mental illness among university students.^9^

It is particularly interesting to consider the links between “little c” creativity and mental health around the transition between childhood and adolescence, as this is a period of pivotal changes in the life trajectory.^10^ Specifically, social and behavioral adjustment at the onset of puberty is associated with a range of psychological, physical, and behavioral outcomes in later life. For example, adjustment at ages 7 and 11 has been found to predict the development of schizophrenia^11^ and malaise^12^ in adulthood, and the chance of developing a long‐standing or limiting illness^12^, ^13^ or chronic widespread pain.^14^ Adjustment has also been found to predict the likelihood of truancy,^15^ offending,^16^ smoking^12^ harmful drinking,^8^ and unemployment^17^ as well as playing a mediating role in the relationship between early life conditions and the age of the first pregnancy in women.^18^ Consequently, there is a need to consider ways of supporting social and behavioral adjustment in childhood.

Research to date could lead us to hypothesize that this “little c” creativity might be beneficial for mental health and adjustment at this transitional age. Creative activities are multimodal pursuits in that they combine many different types of engagement and associated cognitive, behavioral, and emotional responses. For example, creative thinking involves considering abstract terms and adopting multiple viewpoints on an activity. This decentration (the ability to pay attention to multiple aspects of a situation) provided by engagement in creative tasks has been linked with enhanced emotional‐social intelligence, which is defined as an increased ability to understand and express ourselves, understand others, and relate to them, and to cope with daily demands by considering or evaluating consequences of imagined actions.^9^, ^19^ Engaging in creative activities (e.g., drawing, acting, or dancing) encourages persistence and an ability to withstand boredom, which leads to greater self‐discipline and motivation.^5^ It has also been associated with greater self‐acceptance,^20^ which is linked with a lower risk of depression, and encourages both forward planning and the creation of rules that children then follow to manage their own behaviors.^21^ This has been identified as a way of condensing developmental tendencies and therefore directly supporting children's own behavioral development.^22^ Building on this, thinking creatively when engaging in open‐ended tasks can also generate new life paths, helping children to find imaginative ways around potential obstacles.^23^ While some of these same responses can be elicited from other activities (such as sport also leading to greater self‐discipline), it is a success of creative activities in combining multiple different cognitive, behavioral, and emotional responses that provides the theoretical rational for how they might support mental health and positive social and behavioral adjustment in young people. This rationale aligns broadly with literature on the importance of developing noncognitive skills to promote lifetime success in young people.^24^ This study sought to explore this further in children aged 7–11. In defining creativity, we focused on everyday activities that involve engagement in a creative process and draw on imagination,^21^ such as free writing, telling a story, crafts, painting, drawing, or drama.^25^

## Methods

### Participants

We used data from the National Child Development Study (NCDS); a nationally representative British cohort study established in 1958 tracking participants from birth (wave 0).^26^ Specifically, we focused on data from wave 1 (when children were 7 years old) and followed participants up to wave 2 (when children were 11 years old). A total of 12,733 participants had complete data for exposure and outcome. Of these, 917 children were missing data on covariates and were excluded. We further excluded participants who required additional support for developmental delay at school (*n* = 588) and participants who had an upper limb disability that might have affected their ability to engage in creative activities, such as drawing or painting (*n* = 3). This provided an initial sample size of 11,225.

### Measures

Measuring creativity is complex. Performance assessments such as divergent thinking tests have been criticized as too narrow to assess children's engagement in creative processes, while parent or child ratings are considered very subjective.^27^ Ratings by a divergent group of teachers are generally considered a more reliable form of measurement.^28^, ^29^ The NCDS contains teacher self‐reports of creativity for each child whereby teachers are asked to rate the “creativity e.g. in free writing, telling a story, handwork, painting, drawing, dramatic work” of children as either 1 (“shows marked originality or creativity in most areas”), 2 (“usually produces good original work”), 3 (“shows some imagination or originality in most areas”), 4 (“little originality or creativity in all areas), or 5 (“never shows a trace of originality or creativity in any of his work”). While this report relies on the opinion of a single teacher and as such may be subject to bias, studies have found high inter‐ratings between assessments by different teachers.^30^ Further, due to small numbers in the extreme categories, we collapsed categories 1 and 2 together and 4 and 5 together, providing a broader 3‐point variable which we then reverse scored, so that higher scores indicated a higher perceived creativity. While we acknowledge that the nuances of creativity among children may be best captured through multiperson assessments, this method of broad categorization was judged to be suitable for exploring relationships with behavior in this analysis and builds on previous studies that used the same variable from this dataset.^6^, ^7^, ^8^

We measured social and behavioral adjustment using the Bristol Social Adjustment Guide (BSAG).^31^ The BSAG is designed to obtain a picture of a child's behavior in the school setting. It has been used extensively in the NCDS and other research.^32^ Validations comparing teacher assessments with assessments from professional observers, parents, and peers have produced strong positive correlations.^33^ Teachers are asked to underline descriptions that best fit the children they are assessing (e.g., usually friendly/can be surly or suspicious/mumbles shyly, awkwardly/does not answer/answers politely). Researchers then code the items of behaviors that are symptomatic of emotional disturbance or social maladjustment.^32^ These coded items are summed to obtain a quantitative assessment of a child's adjustment at school with higher scores indicating greater adjustment problems. Children with an overall score from 0 to 9 are termed “stable,” while those with a score of 10–19 are termed “unsettled” and those with a score of 20 or more are called “maladjusted.” In our sample, at age 7, 67.3% of children were categorized as stable at baseline, 21.2% as above the threshold for unsettled (a score of 10–19), and 11.4% as above the threshold for maladjusted (a score of 20 or more). Due to the possibility of left‐censoring, whereby participants could enter the study already showing signs of social or behavioral instability or maladjustment which could have affected their engagement in creative activities, we just worked with data from children who were identified as stable at baseline, providing a final sample of 7558 participants.

In addition to an overall score of adjustment, there are also 12 subscales, which group into four categories:^34^ (1) internalizing behaviors (depression, unforthcomingness, writing off of adults and adult standards, and withdrawal); (2) externalizing behaviors (inconsequential behavior such as acting out without regard for others, restlessness, anxiety for acceptance by adults, anxiety for acceptance by children, hostility toward adults, and hostility toward children); (3) miscellaneous nervous symptoms (e.g., getting nervous, blushing, or crying when questioned); and (4) other miscellaneous symptoms (e.g., defensive behaviors). Our primary analysis was of the overall BSAG score and the main four subscale categories^31^: internalizing and externalizing behaviors (which each assessed whether participants had symptoms of instability or maladjustment on any of the individual items within), miscellaneous nervous symptoms, and other miscellaneous symptoms. Sensitivity analyses further explored the results for precise subcomponents of internalizing and externalizing behaviors. Further details about the scoring of these subscales is available as Supplementary Information (available online only).

Given that creativity is thought to be predicted by a number of factors,^35^ our analyses adjusted for a range of potential confounding variables. We relied on parental self‐report for the factors where we identified there to be a low risk of bias in responses. For example, sociodemographic and educational factors were reported by the parents and included gender, social class (using a 5‐class scale based on father's occupation where 1 = the highest status level of social class), poor school attendance (<85% attendance), and educational stability (whether a child had attended more than one school in the past 2 years). We also used parental self‐report as to the presence of mental illness in the family, and whether either parent reads with the child (parent‐reported, rated as neither parent reading weekly, one parent reading weekly, and both parents reading weekly). However, given that parental rating of the time spent with a child could be upwardly biased by parents, we additionally included a rating by the children's teachers as to how much interest mothers showed in their child's schooling (rated by the teacher as little interest, some interest, and very interested). Finally, academic intelligence was measured using scores from the children's performance on the validated psychometric Southgate Group Reading Test and the Problem Arithmetic Test.^36^ For our sensitivity analyses (see below), we also used teachers’ assessments for each child's personality (using teacher‐rated 5‐point scales for cautious/impulsive, mood/even‐tempered, timid/aggressive, flexible/rigid). Personality was measured at wave 3 (when the child was 16) but due to the general perceived stability of personality traits was included in these sensitivity analyses.^37^

### Statistics

To confirm the factor structure of the BSAG, we ran a preliminary factor analysis of the matrix of tetrachoric correlations for all 12 subscales. The Kaiser–Meyer–Olkin measure of sampling adequacy was 0.89 (meritorious). The Kaiser's criterion of eigenvalues > 1 clearly suggested a two‐factor structure, and inspection of a scree plot confirmed this was a reasonable choice. Using both oblique and orthogonal rotations, our data confirmed the previous two‐factor loadings proposed for the BSAG.^34^ Miscellaneous symptoms and miscellaneous nervous symptoms both loaded onto the factor containing *externalizing behaviors*, but with a primary factor loading of <0.4, so they were retained for a separate analysis, also per the original proposals for the BSAG.

We used multinomial logistic regression analyses to calculate the relative risk ratio (RRR) and 95% confidence intervals (CIs) that a child who was categorized as socially and behaviorally stable at age 7 would develop symptoms of social or behavioral instability or maladjustment at age 11. Model 1 adjusted for social, demographic, and educational covariates (gender, social class, school attendance, and educational stability) and family covariates (family mental illness, parental interest in schooling, and parental time reading with the child). Model 2 additionally adjusted for academic ability (reading and mathematics scores). We further used ordinal logistic regression for the fully adjusted models to confirm the direction of trend. In order to ensure representativeness of the sample and analyses and to account for a differential nonresponse, we derived a propensity score for the probability of response at waves 1 and 2 (estimated from a logistic regression model using the covariates from model 2) and weighted all regression models using inverse probability weighting. Fully adjusted models (model 2) are reported below, with the results from model 1 additionally shown in Table 2.

We carried out a series of planned sensitivity analyses. First, we explored whether the inclusion of subthreshold social or behavioral symptoms at baseline affected results by continuing to use just those who were stable at baseline but additionally including their baseline BSAG scores as covariates. Second, we explored whether excluding individuals with instability or maladjustment at baseline may have biased the sample in favor of finding protective effects. Therefore, we reran analyses including those 3667 participants with instability or maladjustment at baseline (Supplementary Table S1, available online only). Third, given known gender differences in social and behavioral maladjustment, we also assessed whether gender was a moderator of the relationship between creativity and adjustment, so we added an interaction term between creativity and gender. Fourth, given studies showing the interconnection between creativity and personality, we explored whether results held when additionally controlling for personality (Supplementary Table S2, available online only). Fifth, we considered that for children who had attended more than one school in the last 2 years, teachers might not be able to make an accurate assessment of their creativity or behaviors. Therefore, we reran analyses excluding those children who had attended two or more schools (18.8%). Finally, in addition to the core analysis of the BSAG scale as a whole and its primary four subcategories, we carried out analyses of all 12 subscales to explore more specifically where differences were found (Supplementary Tables S5A and SB, available online only). All analyses were carried out using Stata SE Version 14.1.

## Results

### Overall symptoms

Demographics of participants are shown in Table 1. When working just with children who were identified as socially and behaviorally stable at age 7, 15.8% went on to become unsettled by age 11 (a score of 10–19) and 6.1% to become maladjusted (a score of 20‐max).

**Table 1:** Demographic profile of participants included in analyses

	N (%)
Male, *n* (%)	4090 (54.1)
Social class	
I	440 (5.08)
II	1210 (16.0)
III	4120 (54.5)
IV	1229 (16.3)
V	559 (7.4)
Attended >1 school in last 2 years	1417 (18.8)
Poor school attendance (<85%)	1205 (15.9)
Mental illness in the family	164 (2.2)
Maternal interest in schooling	
Little interest	1104 (14.6)
Some interest	2914 (38.6)
Very interested	3540 (46.8)
Parent reads to child weekly	
Neither parent	3260 (43.1)
One parent	2042 (27.0)
Both parents	2256 (29.9)
Reading score /30, μ (SD)	25.6 (5.4)
Maths score /10, μ (SD)	5.7 (2.3)

In comparison to children who were identified as showing little creativity, children who showed some creativity at age 7 had a 22% lower relative risk of social and behavioral instability at age 11 (RRR = 0.78, 95% CI: 0.66–0.92, *P* = 0.003), and children who showed marked creativity had a 36% lower relative risk even when adjusting for all identified confounding variables (RRR = 0.64, 95% CI: 0.52–0.79, *P* < 0.001). Additionally, showing some creativity at age 7 was associated with a 31% lower relative risk of social and behavioral maladjustment at age 11 (RRR = 0.69, 95% CI: 0.54–0.88, *P* = 0.002), and showing marked creativity was associated with a 49% lower relative risk even when adjusting for all identified confounding variables (RRR = 0.51, 95% CI: 0.37–0.71, *P* < 0.001) (Table 2).

**Table 2:** Associations of creativity with symptoms of social and behavioral instability and maladjustment

		Model 1			Model 2			
		RRR	*P*	95% CI	RRR	*P*	95% CI	*P* for trend
**OVERALL**								
** **Symptoms of instability	Little creativity	REF	REF	REF	REF	REF	REF	
	Some creativity	**0.69** *	**<0.001** *	**0.59–0.81** *	**0.78** *	**0.003** *	**0.66–0.92** *	Little creativity: REF
	Marked creativity	**0.52** *	**<0.001** *	**0.43–0.63** *	**0.64** *	**<0.001** *	**0.52–0.79** *	**Some creativity**: **<0.001** *
** **Symptoms of maladjustment	Little creativity	REF	REF	REF	REF	REF	REF	**Marked creativity: <0.001** *
	Some creativity	**0.54** *	**<0.001** *	**0.43–0.67** *	**0.69** *	**0.002** *	**0.54–0.88** *	
	Marked creativity	**0.34** *	**<0.001** *	**0.25–0.46** *	**0.51** *	**<0.001** *	**0.37–0.71** *	
**SUBSCALES**								
**Internalizing behaviors**								
** **Symptoms of instability	Little creativity	REF	REF	REF	REF	REF	REF	
	Some creativity	**0.70** *	**<0.001** *	**0.60–0.81** *	**0.74** *	**<0.001** *	**0.64–0.87** *	Little creativity: REF
	Marked creativity	**0.58** *	**<0.001** *	**0.49 –0.69** *	**0.65** *	**<0.001** *	**0.54–0.78** *	**Some creativity: <0.001** *
** **Symptoms of maladjustment	Little creativity	REF	REF	REF	REF	REF	REF	**Marked creativity: <0.001** *
	Some creativity	**0.53** *	**<0.001** *	**0.44–0.63** *	**0.64** *	**<0.001** *	**0.53–0.76** *	
	Marked creativity	**0.33** *	**<0.001** *	**0.27–0.41** *	**0.46** *	**<0.001** *	**0.36–0.57** *	
**Externalizing behaviors**								
** **Symptoms of instability	Little creativity	REF	REF	REF	REF	REF	REF	
	Some creativity	**0.86** *	**0.036** *	**0.75 –0.99** *	0.95	0.48	0.82–1.10	Little creativity: REF
	Marked creativity	**0.76** *	**0.001** *	**0.65–0.90** *	0.90	0.22	0.76–1.07	**Some creativity: 0.029** *
** **Symptoms of maladjustment	Little creativity	REF	REF	REF	REF	REF	REF	**Marked creativity: 0.007** *
	Some creativity	**0.69** *	**<0.001** *	**0.58–0.82** *	**0.81** *	**0.022** *	**0.68–0.97** *	
	Marked creativity	**0.58** *	**<0.001** *	**0.47–0.71** *	**0.75** *	**0.008** *	**0.60–0.93** *	
**Miscellaneous symptoms**								
** **Symptoms of instability	Little creativity	REF	REF	REF	REF	REF	REF	
	Some creativity	**0.68** *	**<0.001** *	**0.58–0.79** *	**0.74** *	**<0.001** *	**0.63–0.87** *	Little creativity: REF
	Marked creativity	**0.58** *	**<0.001** *	**0.48–0.70** *	**0.67** *	**<0.001** *	**0.55–0.81** *	**Some creativity: <0.001** *
** **Symptoms of maladjustment	Little creativity	REF	REF	REF	REF	REF	REF	**Marked creativity: <0.001** *
	Some creativity	**0.59** *	**<0.001** *	**0.49–0.71** *	**0.69** *	**<0.001** *	**0.57–0.84** *	
	Marked creativity	**0.42** *	**<0.001** *	**0.33–0.53** *	**0.55** *	**<0.001** *	**0.42–0.71** *	
**Miscellaneous nervous symptoms**								
** **Symptoms of instability	Little creativity	REF	REF	REF	REF	REF	REF	
	Some creativity	**0.78** *	**0.027** *	**0.62–0.97** *	0.85	0.17	0.67–1.07	Little creativity: REF
	Marked creativity	**0.52** *	**<0.001** *	**0.39 –0.70** *	**0.61** *	**0.002** *	**0.44–0.83** *	Some creativity: 0.17
** **Symptoms of maladjustment	Little creativity	REF	REF	REF	REF	REF	REF	**Marked creativity: 0.002** *
	Some creativity	**–**	**–**	**–**	**–**	**–**	**–**	
	Marked creativity	**–**	**–**	**–**	**–**	**–**	**–**	

^*^ *P* < 0.05 is in bold font.

REF: stable. Model 1 adjusted for social, demographic, and educational covariates (sex, social class, school attendance, and educational stability) and family covariates (family mental illness, parental interest in schooling, and parental time reading with the child). Model 2 additionally adjusted for academic ability.

### Internalizing behavior symptoms

In comparison to children who were identified as showing little creativity, children who showed some creativity at age 7 had a 26% lower relative risk of symptoms of instability in internalizing behaviors at age 11 (RRR = 0.74, 95% CI: 0.64–0.87, *P* <0.001), and children who showed marked creativity had a 35% lower relative risk even when adjusting for all identified confounding variables (RRR = 0.65, 95% CI: 0.54–0.78, *P* < 0.001). Additionally, showing some creativity at age 7 was associated with a 36% lower relative risk of symptoms of maladjustment in internalizing behaviors at age 11 (RRR = 0.64, 95% CI: 0.53–0.76, *P* < 0.001), and showing marked creativity was associated with a 54% lower relative risk even when adjusting for all identified confounding variables (RRR = 0.46, 95% CI: 0.36–0.57, *P* < 0.001) (Table 2).

Sensitivity analyses using the four subscales of internalizing behaviors showed that, in comparison to children who were identified as showing little creativity, showing some or marked creativity at age 7 was associated with a lower risk of symptoms of instability for depression, unforthcomingness, and a tendency to write off adults, but not for withdrawal. Showing some or marked creativity at age 7 was associated with a lower risk of symptoms of maladjustment for all four subscales (depression, unforthcomingness, a tendency to write off adults, and withdrawal) (Supplementary Table S5A, available online only).

### Externalizing behavior symptoms

In comparison with children who were identified as showing little creativity, children who showed some creativity at age 7 did not have a lower relative risk of symptoms of instability at age 11 once intelligence was taken into account (RRR = 0.95, 95% CI: 0.82–1.10, *P* = 0.48), and nor did children who showed marked creativity (RRR = 0.90, 95% CI: 0.76–1.07, *P* = 0.22). However, showing some creativity at age 7 was associated with a 19% lower relative risk of symptoms of maladjustment at age 11 (RRR = 0.81, 95% CI: 0.68–0.97, *P* = 0.022), and showing marked creativity was associated with a 25% lower relative risk even when adjusting for all identified confounding variables (RRR = 0.75, 95% CI: 0.60–0.93, *P* = 0.008). (Table 2).

Sensitivity analyses using the six subscales of externalizing behaviors showed that, in comparison to children who were identified as showing little creativity, showing some or marked creativity at age 7 was associated with a lower risk of symptoms of instability for inconsequential behaviors and restlessness, but not for anxiety to be accepted either by adults or children, or hostility toward either adults or children. Showing some or marked creativity at age 7 was associated with a lower risk of symptoms of maladjustment for just inconsequential behaviors (Supplementary Table S5B, available online only).

### Miscellaneous behavior symptoms

For miscellaneous symptoms, in comparison to children who were identified as showing little creativity, children who showed some creativity at age 7 had a 26% lower relative risk of symptoms of instability in miscellaneous symptoms at age 11 (RRR = 0.74, 95% CI: 0.63–0.87, *P* < 0.001), and children who showed marked creativity had a 33% lower relative risk even when adjusting for all identified confounding variables (RRR = 0.67, 95% CI: 0.55–0.81, *P* < 0.001). Showing some creativity at age 7 was associated with a 31% lower relative risk of symptoms of maladjustment in internalizing behaviors at age 11 (RRR = 0.69, 95% CI: 0.57–0.84, *P* < 0.001), and showing marked creativity was associated with a 45% lower relative risk even when adjusting for all identified confounding variables (RRR = 0.55, 95% CI: 0.42–0.71, *P* < 0.001) (Table 2).

For miscellaneous nervous symptoms, in comparison to children who were identified as showing little creativity, children who showed some creativity at age 7 did not have a lower relative risk of symptoms of instability at age 11 (RRR = 0.85, 95% CI: 0.67–1.07, *P* = 0.17). But children who showed marked creativity had a 39% lower relative risk of symptoms of instability even when adjusting for all identified confounding variables (RRR = 0.61, 95% CI: 0.44–0.83, *P* = 0.002). No threshold was available to measure maladjustment in miscellaneous nervous symptoms.

### Further sensitivity analyses

In addition to the subscale sensitivity analyses, further sensitivity analyses showed that the inclusion of the precise baseline BSAG score as well as just including participants who were categorized as stable did not lead to an attenuation of results (Supplementary Table S1, available online only). Further, our decision to exclude those with instability or maladjustment at baseline did not bias results toward protective effects. Indeed, when including all individuals at baseline, significant results were replicated more strongly (Supplementary Table S2, available online only). When exploring gender differences, inclusion of an interaction term in the analyses revealed no gender differences for overall BSAG score. Moreover, the inclusion of personality as a further covariate did not attenuate any results (Supplementary Table S3, available online only). Finally, the exclusion of children who attended more than one school in the last 2 years did not materially affect overall results, although the results for externalizing behaviors were attenuated (Supplementary Table S4, available online only).

## Discussion

This study is the first to explore longitudinal associations between creativity (defined in this instance as imagination in undertaking activities, such as free writing, telling a story, crafts, painting, drawing, or drama) and adjustment in children. We found that creativity at age 7 among children who were free from social or behavioral adjustment issues was associated with a lower relative risk of social and behavioral instability and maladjustment at age 11. Specifically, associations were found with a lower risk of symptoms of instability of internalizing behaviors (including depression, unforthcomingness, and an attitude of writing off adults), externalizing behaviors (including inconsequential behaviors and restlessness), miscellaneous symptoms, and other miscellaneous nervous symptoms. Additionally, associations were found with a lower risk of symptoms of maladjustment of internalizing behaviors (including depression, unforthcomingness, an attitude of writing off adults, and withdrawal), externalizing behaviors (inconsequential behavior), and other miscellaneous symptoms. Notably, covariates relating to the academic ability (model 2) explained some of the association, reducing the strength of associations by 20–30% and attenuating results completely for the symptoms of instability in externalizing behaviors (present in model 1 but not model 2). However, the remainder of the results were maintained independently of social, demographic, educational, parental, academic, and personality covariates, suggesting that results are not merely a function of these other individual and social factors.

This study has a number of strengths. It drew participants from a large and nationally representative sample that includes repeated measures of behavior, collected prospectively during childhood. The large number of variables within the NCDS meant that we were able to control for all identified confounding variables, including socioeconomic status, home environment, personality, and intelligence that have been shown in previous studies to covary with measures of creativity. We also relied on assessments of creativity and behaviors made by a third party (teachers) rather than on self‐report. However, this study also had some limitations. Definitions of creativity vary and are not immutable, nor universally accepted.^38^, ^39^, ^40^ However, as with other complex constructs, such as personality and intelligence, while this might pose challenges for measurements, it should not be a barrier to research, so we followed well‐accepted definitions. Further, despite differences in measurements of creativity, there has to date been a high degree of convergence between the results using different measures.^35^ Therefore, in this study, we focused on creativity when undertaking creative activities and used ratings by teachers. While there is a certain amount of subjectivity in such ratings, these ratings were limited to a simple 5‐point scale to minimize options (later collapsed by us into 3 points), and we used measurements by teachers rather than parents to gain a more objective measure. Further, this measurement is recognized as having the strength that teachers can take into account a child's creativity as demonstrated in a range of activities over a period of time, providing a comprehensive rather than moment‐specific assessment,^41^ and has been used previously in a number of studies as cited above. Similarly, teachers were also able to consider children's behavior within the context of their class, which means that all observations could be benchmarked against a broader distribution of behaviors. This benchmarking is particularly important given that our analyses focused on the period from childhood to puberty, which is recognized as a time of transition and therefore likely to involve natural changes in a child's behavior anyway.^10^ However, it is recognized that a teacher might have been working in a school that had, for example, highly creative children (leading to a down‐estimation of a target child's creativity). Therefore, future studies might need to include measures of the classroom environments in which the creative activities being assessed were carried out, given that the environment has been shown to foster creative development,^42^ and explore how developments in the classroom environments and approaches to teaching since these data were gathered might be affecting creative participation in children. Also, as the population of this study was largely homogeneous White British children, reflecting the national demographic at the time of data collection, it would be relevant to consider through the future research whether the same findings can be generalized to different cultural backgrounds.

In light of the results herein, it is relevant to consider how creativity could be supported among primary school children. Research into the environmental influences of creativity focuses on what sorts of *climates* support creativity.^35^ The systems view of creativity proposes that, although most focus within creativity research has been on the individual, creative acts are embedded within the society and culture, and as such the institutions within society need to enable creativity.^43^ Further, the development of creativity is not static but is made up of ongoing dynamic reorganizations.^44^ As a result, there has been a call for creativity to be encouraged and supported in everyday life in schools.^42^, ^45^ In particular, studies have demonstrated the value of teacher encouraged creativity and intergenerational mentoring,^3^ not as a way of creating “Big C” highly creative individuals, but more to encourage the individual and behavioral processes inherent in creativity as a way of contributing to the maintenance of mental health.^46^

In conclusion, creativity when engaging in activities such as free writing, telling a story, handwork, painting, drawing, and dramatic work in primary‐aged children is associated with a reduced relative risk of social and behavioral instability at the onset of adolescence. Further work remains to be undertaken to explore whether facilitating engagement with creative activities could therefore be used as an intervention to reduce levels of instability and maladjustment, thereby removing a risk factor for the development of further psychological or physical health conditions or health‐impairing behaviors later in life.

## Competing interests

The authors declare no competing interests.

## Supporting information


**Supplementary Table 1**. Associations between creativity and overall symptoms of social and behavioral instability or maladjustment when additionally adjusting for baseline BSAG score.

**Supplementary Table 2**. Associations between creativity and overall symptoms of social and behavioral instability or maladjustment when including those with baseline maladjustment.

**Supplementary Table 3**. Associations between creativity and overall symptoms of social and behavioral instability or maladjustment when including personality as an additional covariate.

**Supplementary Table 4**. Associations between creativity and overall symptoms of social and behavioral instability or maladjustment when excluding participants who had attended more than one school in the past 2 years.

**Supplementary Table 5A**. Associations between creativity and symptoms relating to internalizing behaviors.

**Supplementary Table 5B**. Associations between creativity and symptoms relating to externalizing behaviors.

**Supporting Information File 1**
