# Supplementary material for: Effects of creativity on social and behavioral adjustment in 7‐ to 11‐year‐old children
Source: Ann N Y Acad Sci. 2018 Aug 5;1438(1):30–9. doi: 10.1111/nyas.13944 (PMC6446801; doi:10.1111/nyas.13944)
Supplement: Supplementary file 1 — Supplementary Table 1. Associations between creativity and overall symptoms of social and behavioral instability or maladjustment when additionally adjusting for baseline BSAG score. [file NYAS-1438-30-s001.docx]

**Supplementary Table 1.** Associations between creativity and overall symptoms of social and behavioral instability or maladjustment when additionally adjusting for baseline BSAG score.

|  |  | **Model 2** | | |
| --- | --- | --- | --- | --- |
|  |  | **RRR** | **p** | **95% CI** |
| **OVERALL** | | | | |
| Symptoms of instability | Little creativity | REF | REF | REF |
|  | Some creativity | 0.85 | .053 | 0.72–1.00 |
|  | Marked creativity | **0.71** | **.002** | **0.58–0.88** |
| Symptoms of maladjustment | Little creativity | REF | REF | REF |
|  | Some creativity | 0.80 | .067 | 0.62–1.02 |
|  | Marked creativity | **0.60** | **.004** | **0.43–0.85** |
| **SUBSCALES**  **Internalizing behaviors** | | | | |
| Symptoms of instability | Little creativity | REF | REF | REF |
|  | Some creativity | **0.80** | **.006** | **0.68–0.94** |
|  | Marked creativity | **0.71** | **<.001** | **0.59–0.85** |
| Symptoms of maladjustment | Little creativity | REF | REF | REF |
|  | Some creativity | **0.73** | **.001** | **0.61–0.88** |
|  | Marked creativity | **0.54** | **<.001** | **0.43–0.67** |
| **Externalizing behaviors** | | | | |
| Symptoms of instability | Little creativity | REF | REF | REF |
|  | Some creativity | 0.95 | .50 | 0.82–1.10 |
|  | Marked creativity | 0.90 | .23 | 0.76–1.07 |
| Symptoms of maladjustment | Little creativity | REF | REF | REF |
|  | Some creativity | **0.81** | **.023** | **0.68–0.97** |
|  | Marked creativity | **0.75** | **.009** | **0.60–0.93** |
| **Miscellaneous symptoms** | | | | |
| Symptoms of instability | Little creativity | REF | REF | REF |
|  | Some creativity | **0.76** | **.001** | **0.65–0.89** |
|  | Marked creativity | **0.70** | **<.001** | **0.57–0.85** |
| Symptoms of maladjustment | Little creativity | REF | REF | REF |
|  | Some creativity | **0.72** | **.001** | **0.59–0.87** |
|  | Marked creativity | **0.58** | **<.001** | **0.45–0.75** |
| **Miscellaneous nervous symptoms** | | | | |
| Symptoms of instability | Little creativity | REF | REF | REF |
|  | Some creativity | 0.85 | .19 | 0.67–1.08 |
|  | Marked creativity | **0.61** | **.002** | **0.45–0.84** |
| Symptoms of maladjustment | Little creativity | REF | REF | REF |
|  | Some creativity | **-** | **-** | **-** |
|  | Marked creativity | **-** | **-** | **-** |

Note: N=7,558. REF: stable. Model 2 adjusted for social, demographic and educational covariates (sex, social class, school attendance and educational stability) and family covariates (family mental illness, parental interest in schooling and parental time reading with the child) and academic ability as well as baseline BSAG score.
