# Supplementary material for: Effects of creativity on social and behavioral adjustment in 7‐ to 11‐year‐old children
Source: Ann N Y Acad Sci. 2018 Aug 5;1438(1):30–9. doi: 10.1111/nyas.13944 (PMC6446801; doi:10.1111/nyas.13944)
Supplement: Supplementary file 5 — Supplementary Table 5A. Associations between creativity and symptoms relating to internalizing behaviors. [file NYAS-1438-30-s005.docx]

**Supplementary Table 5A:** Associations between creativity and symptoms relating to internalizing behaviors.

|  |  | **Model 1** | | | **Model 2** | | | **P for trend** |
| --- | --- | --- | --- | --- | --- | --- | --- | --- |
|  |  | **RRR** | **p** | **95% CI** | **RRR** | **p** | **95% CI** |  |
| **Depression** | | | | | | | | |
| Symptoms of instability | Little creativity | REF | REF | REF | REF | REF | REF |  |
|  | Some creativity | **0.74** | **<.001** | **0.64–0.85** | **0.83** | **.012** | **0.72–0.96** | **Some creativity: <.001**  **Marked creativity: <.001** |
|  | Marked creativity | **0.61** | **<.001** | **0.51–0.72** | **0.74** | **.001** | **0.62–0.88** |  |
| Symptoms of maladjustment | Little creativity | REF | REF | REF | REF | REF | REF |  |
|  | Some creativity | **0.51** | **<.001** | **0.42–0.62** | **0.62** | **<.001** | **0.51–0.76** |  |
|  | Marked creativity | **0.33** | **<.001** | **0.26–0.43** | **0.46** | **<.001** | **0.35–0.61** |  |
| **Unforthcomingness** | | | | | | | | |
| Symptoms of instability | Little creativity | REF | REF | REF | REF | REF | REF |  |
|  | Some creativity | **0.69** | **<.001** | **0.61–0.79** | **0.72** | **<.001** | **0.63–0.82** | **Some creativity: <.001**  **Marked creativity: <.001** |
|  | Marked creativity | **0.65** | **<.001** | **0.56–0.75** | **0.70** | **<.001** | **0.60–0.82** |  |
| Symptoms of maladjustment | Little creativity | REF | REF | REF | REF | REF | REF |  |
|  | Some creativity | **0.58** | **<.001** | **0.47–0.73** | **0.67** | **.001** | **0.54–0.85** |  |
|  | Marked creativity | **0.36** | **<.001** | **0.27–0.48** | **0.47** | **<.001** | **0.35–0.64** |  |
| **Writing off adults** | | | | | | | | |
| Symptoms of instability | Little creativity | REF | REF | REF | REF | REF | REF |  |
|  | Some creativity | **0.78** | **.001** | **0.68–0.90** | **0.85** | **.033** | **0.74–0.99** | **Some creativity: .005**  **Marked creativity: <.001** |
|  | Marked creativity | **0.69** | **<.001** | **0.58–0.81** | **0.78** | **.006** | **0.66–0.93** |  |
| Symptoms of maladjustment | Little creativity | REF | REF | REF | REF | REF | REF |  |
|  | Some creativity | **0.66** | **<.001** | **0.54–0.80** | **0.79** | **.024** | **0.64–0.97** |  |
|  | Marked creativity | **0.40** | **<.001** | **0.31–0.53** | **0.54** | **<.001** | **0.41–0.72** |  |
| **Withdrawal** | | | | | | | | |
| Symptoms of instability | Little creativity | REF | REF | REF | REF | REF | REF |  |
|  | Some creativity | 0.88 | .19 | 0.72–1.07 | 0.94 | .54 | 0.77–1.15 | Some creativity: .091  Marked creativity: .091 |
|  | Marked creativity | 0.90 | .34 | 0.71–1.12 | 0.99 | .95 | 0.78–1.26 |  |
| Symptoms of maladjustment | Little creativity | REF | REF | REF | REF | REF | REF |  |
|  | Some creativity | **0.69** | **.002** | **0.54–0.88** | 0.78 | .06 | 0.61–1.01 |  |
|  | Marked creativity | **0.48** | **<.001** | **0.35–0.67** | **0.61** | **.005** | **0.43–0.86** |  |

Note: REF: stable. Model 1 adjusted for social, demographic and educational covariates (sex, social class, school attendance and educational stability) and family covariates (family mental illness, parental interest in schooling and parental time reading with the child). Model 2 additionally adjusted for academic ability.
