# Supplementary material for: Effects of creativity on social and behavioral adjustment in 7‐ to 11‐year‐old children
Source: Ann N Y Acad Sci. 2018 Aug 5;1438(1):30–9. doi: 10.1111/nyas.13944 (PMC6446801; doi:10.1111/nyas.13944)
Supplement: Supplementary file 6 — Supplementary Table 5B. Associations between creativity and symptoms relating to externalizing behaviors. [file NYAS-1438-30-s006.docx]

**Supplementary Table 5B.** Associations between creativity and symptoms relating to externalizing behaviors.

|  |  | **Model 1** | | | **Model 2** | | | **P for trend** |
| --- | --- | --- | --- | --- | --- | --- | --- | --- |
|  |  | **RRR** | **p** | **95% CI** | **RRR** | **p** | **95% CI** |  |
| **Inconsequential behavior** | | | | | | | | |
| Symptoms of instability | Little creativity | REF | REF | REF | REF | REF | REF |  |
|  | Some creativity | **0.82** | **.003** | **0.72–0.93** | **0.91** | **.18** | **0.79–1.04** | **Some creativity: .006**  **Marked creativity: <.001** |
|  | Marked creativity | **0.67** | **<.001** | **0.57–0.78** | **0.80** | **.007** | **0.68–0.94** |  |
| Symptoms of maladjustment | Little creativity | REF | REF | REF | REF | REF | REF |  |
|  | Some creativity | **0.55** | **<.001** | **0.44–0.67** | **0.71** | **.003** | **0.57–0.89** |  |
|  | Marked creativity | **0.30** | **<.001** | **0.23–0.41** | **0.46** | **<.001** | **0.34–0.63** |  |
| **Restlessness** | | | | | | | | |
| Symptoms of instability | Little creativity | REF | REF | REF | REF | REF | REF |  |
|  | Some creativity | **0.66** | **<.001** | **0.56–0.79** | **0.79** | **.009** | **0.66–0.94** |  |
|  | Marked creativity | **0.35** | **<.001** | **0.28–0.45** | **0.46** | **<.001** | **0.36–0.59** |  |
| Symptoms of maladjustment | Little creativity | REF | REF | REF | REF | REF | REF |  |
|  | Some creativity | **-** | **-** | **-** | **-** | **-** | **-** |  |
|  | Marked creativity | **-** | **-** | **-** | **-** | **-** | **-** |  |
| **Anxiety for acceptance by adults** | | | | | | | | |
| Symptoms of instability | Little creativity | REF | REF | REF | REF | REF | REF |  |
|  | Some creativity | 0.89 | .17 | 0.76–1.05 | 0.96 | .60 | 0.81–1.13 | Some creativity: .78  Marked creativity: .51 |
|  | Marked creativity | 0.86 | .12 | 0.72–1.04 | 0.96 | .69 | 0.79–1.17 |  |
| Symptoms of maladjustment | Little creativity | REF | REF | REF | REF | REF | REF |  |
|  | Some creativity | 0.95 | .65 | 0.76–1.18 | 1.01 | .91 | 0.81–1.27 |  |
|  | Marked creativity | 0.84 | .19 | 0.65–1.09 | 0.92 | .53 | 0.69–1.21 |  |
| **Anxiety for acceptance by children** | | | | | | | | |
| Symptoms of instability | Little creativity | REF | REF | REF | REF | REF | REF |  |
|  | Some creativity | **0.81** | **.035** | **0.66–0.99** | 0.86 | .16 | 0.71–1.06 | Some creativity: .35  Marked creativity: .78 |
|  | Marked creativity | 0.85 | .15 | 0.67–1.06 | 0.92 | .52 | 0.72–1.18 |  |
| Symptoms of maladjustment | Little creativity | REF | REF | REF | REF | REF | REF |  |
|  | Some creativity | 0.98 | .90 | 0.74–1.30 | 1.03 | .86 | 0.77–1.37 |  |
|  | Marked creativity | 0.99 | .97 | 0.72–1.37 | 1.06 | .75 | 0.75–1.49 |  |
| **Hostility towards adults** | | | | | | | | |
| Symptoms of instability | Little creativity | REF | REF | REF | REF | REF | REF |  |
|  | Some creativity | 1.02 | .85 | 0.86–1.21 | 1.09 | .32 | 0.92–1.31 | Some creativity: .85  Marked creativity: .48 |
|  | Marked creativity | 0.85 | .12 | 0.69–1.04 | 0.96 | .69 | 0.77–1.19 |  |
| Symptoms of maladjustment | Little creativity | REF | REF | REF | REF | REF | REF |  |
|  | Some creativity | 0.86 | .17 | 0.70–1.07 | 0.94 | .60 | 0.75–1.18 |  |
|  | Marked creativity | 0.82 | .13 | 0.63–1.06 | 0.93 | .63 | 0.71–1.23 |  |
| **Hostility towards children** | | | | | | | | |
| Symptoms of instability | Little creativity | REF | REF | REF | REF | REF | REF |  |
|  | Some creativity | 0.88 | .17 | 0.74–1.06 | 0.98 | .85 | 0.81–1.19 |  |
|  | Marked creativity | **0.75** | **.011** | **0.61–0.94** | 0.89 | .33 | 0.71–1.12 |  |
| Symptoms of maladjustment | Little creativity | REF | REF | REF | REF | REF | REF |  |
|  | Some creativity | - | - | - | - | - | - |  |
|  | Marked creativity | - | - | - | - | - | - |  |

REF: stable. Model 1 adjusted for social, demographic and educational covariates (sex, social class, school attendance and educational stability) and family covariates (family mental illness, parental interest in schooling and parental time reading with the child). Model 2 additionally adjusted for academic ability.
