# Supplementary material for: Effects of creativity on social and behavioral adjustment in 7‐ to 11‐year‐old children
Source: Ann N Y Acad Sci. 2018 Aug 5;1438(1):30–9. doi: 10.1111/nyas.13944 (PMC6446801; doi:10.1111/nyas.13944)
Supplement: Supplementary file 7 — Supporting Information File 1 [file NYAS-1438-30-s007.docx]

**Supplementary Information**

When working with the subscales of the Bristol Social Adjustment Guide (BSAG), there are no equivalent cut offs to those available for the overall BSAG score. However, the strong positive skew of the distribution requires the categorisation of scores.

Previous analyses have proposed a “stable” category as equating to the lowest 50% of children and a “maladjustment” category as equating to the top 13% of children for each subscale, with those in the middle categorised as “unstable” (1). Consequently, we used this as our guide, maintaining the same 3-level structure, but proposed slightly different cut-offs.

Instead of categorising the lowest 50% as stable, we used a score of “0” for each subscale as an indication of being stable. This decision was made as a score of 0 was felt to be a clearer indication of stability than children falling in a particular percentile, and also because the positive skew of all subscales meant that more than 50% of children scored 0 on most of the subscales (range 49.2% and 93.7%; see table below), so it was not possible to select just the lowest 50%.

Instead of categorizing the top 13% as maladjusted, we used the more conservative top 10% (which provided an actual range of 4.5–9.2% across the different subscales). This more conservative percentage was chosen to make a clearer distinction between the presence of just 1 or 2 symptoms of instability vs the presence of multiple symptoms.

The precise scores for each subscale and percentage of children scoring in each category are provided below:

**Cut offs used for subscales of the Bristol Social Adjustment Guide (BSAG)**

|  | **“Stable”** | | **“Symptoms of instability”** | | **“Maladjusted”** | |
| --- | --- | --- | --- | --- | --- | --- |
|  | Score | Percentage | Score | Percentage | Score | Percentage |
| Depression | 0 | 56.6% | 1–2 | 34.5% | 3+ | 8.9% |
| Unforthcomingness | 0 | 43.9% | 1–4 | 49.0% | 5+ | 7.1% |
| Writing off of adults | 0 | 58.9% | 1–2 | 32.5% | 3+ | 8.6% |
| Withdrawal | 0 | 80.4% | 1 | 15.1% | 2+ | 4.5% |
| **Total**  **= Internalizing behaviors** |  | **35.2%** |  | **46.1%** |  | **18.8%** |
|  |  |  |  |  |  |  |
| Inconsequential behaviors | 0 | 49.2% | 1–3 | 44.0% | 4+ | 6.8% |
| Restlessness | 0 | 89.5% | 1+ | 10.5% | -^a^ | - |
| Anxiety for acceptance by adults | 0 | 66.2% | 1 | 25.5% | 2+ | 8.3% |
| Anxiety for acceptance by children | 0 | 81.2% | 1 | 14.2% | 2+ | 4.6% |
| Hostility towards adults | 0 | 70.3% | 1–2 | 22.2% | 3+ | 7.5% |
| Hostility towards children | 0 | 89.2% | 1+ | 10.8% | - ^a^ | - |
| **Total**  **= Externalizing behaviors** |  | **39.0%** |  | **44.3%** |  | **16.7%** |
|  |  |  |  |  |  |  |
| **Miscellaneous symptoms** | 0 | 64.6% | 1 | 26.2% | 2+ | 9.2% |
| **Miscellaneous nervous symptoms** | 0 | 93.7% | 1+ | 6.3% | - ^a^ | - |
| ^a^There were no cut-offs higher than the threshold for ‘symptoms of instability’ large enough to carry out analyses. | | | | | | |
